# Psychometric Properties of the Pre-Literacy Test: Assessing Literacy Readiness Skills

**DOI:** 10.3390/jintelligence13120155

**Published:** 2025-12-02

**Authors:** Muhammet Baştuğ

**Affiliations:** Faculty of Education, Primary Education, Yildiz Technical University, Istanbul 34220, Türkiye; muhammet.bastug@yildiz.edu.tr

**Keywords:** early literacy, literacy skills, test development, psychometric analysis

## Abstract

This study examined the psychometric properties of the Pre-Literacy Test, developed to measure the literacy readiness skills of children who have completed preschool education. Using a quantitative, multistage design, the study was conducted with a total of 5966 children aged 6–7 who were about to enter elementary school in the 2024–2025 academic year (N_1_ = 1911; N_2_ = 1644; N_3_ = 2411). Exploratory Factor Analysis revealed a three-factor structure—Reading Skills, Writing Skills (Dictation), and Writing Skills (Copying)—which explained 82.38% of the total variance. Confirmatory Factor Analysis demonstrated that this structure showed an acceptable model fit (CFI = 0.997, TLI = 0.997, SRMR = 0.030, RMSEA = 0.111). The internal consistency coefficients (α = 0.891–0.962; ω = 0.912–0.983) and convergent validity values (AVE = 0.867–0.949) of the PLT were found to be high. Discriminant validity was confirmed according to the Fornell–Larcker criterion, and measurement invariance across gender was supported through Multigroup Confirmatory Factor Analysis. Item analyses indicated that most test items were of moderate difficulty (mean difficulty = 0.409) and high discrimination (mean discrimination = 0.516). In conclusion, the PLT was determined to be a psychometrically robust, valid, and reliable instrument for assessing basic literacy skills prior to elementary school entry. These findings suggest that the test can be confidently used in early literacy research and school readiness assessments.

## 1. Introduction

Early literacy skills are among the most critical skills that determine a child’s educational achievement and overall quality of life. These skills represent a multidimensional network of interrelated cognitive competencies, ranging from basic letter recognition to phonological processing, rapid naming, print awareness, and oral language proficiency (Ne’eman and Shaul 2023). Early literacy develops through a dynamic process that encompasses all the interactions and awareness children have with written and spoken language in their environment before they receive formal reading and writing instruction (Bonifacci et al. 2022). Subcomponents such as phonological awareness, alphabet knowledge, print awareness, and vocabulary acquired during this period serve as a strong prerequisite for the development of fundamental literacy skills that begin in elementary school (Pham et al. 2025).

Early literacy skills encompass the knowledge, skills, and attitudes that children must acquire before reading and writing, including critical components such as phonological awareness (recognizing, discriminating, and manipulating sounds), letter knowledge, vocabulary, and print awareness (understanding how books and print function) (Derby et al. 2020; Ergin et al. 2025). Acquiring these skills during early childhood has significant and long-term effects on an individual’s cognitive, socio-emotional, and academic development. In particular, phonological awareness has been found to directly enhance reading success, expand vocabulary through reading activities, strengthen grammatical skills, and improve oral expression (De Abreu et al. 2020). Moreover, reading experiences support higher-order cognitive processes such as attention, memory, problem-solving, and planning, thereby contributing to the development of executive functions (Blair and Raver 2014; Ne’eman and Shaul 2023). Children who develop literacy skills at an early age demonstrate greater success in reading and comprehension skills in later years (Inoue et al. 2023) and facilitate learning in other academic areas by enhancing their overall school achievement (Pham et al. 2025). Research has also shown that children with this strong foundation are more likely to succeed in school, pursue further education, and are less likely to drop out (Hernandez 2011).

Neuroscientific research demonstrates that early literacy experiences have positive effects on brain development and strengthen the neural connections among regions associated with reading, enabling these skills to become faster and more automatic (Benischek et al. 2020; Mateu-Estivill et al. 2020). Strong early literacy skills positively influence an individual’s academic success not only in elementary school but also in middle and high school, as well as their access to better educational opportunities and upward social mobility later in life (Shahaeian et al. 2018). Moreover, sufficient reading skills contribute to children’s social and emotional development by enhancing their self-confidence and fostering a positive attitude toward learning; children with strong reading and social skills exhibit better academic performance (Carpendale et al. 2025; Cooper et al. 2014). Conversely, children with reading difficulties are more likely to experience attention deficits, poor self-regulation, and problems with social adjustment resulting from their learning difficulties (Montroy et al. 2014). The lack of reading skills is strongly associated with academic failure and can negatively affect children’s self-esteem, sense of self-efficacy, and overall psychological well-being, leading to feelings of inadequacy (Humphrey 2003; Ne’eman and Shaul 2023). Such individuals have been found to display lower academic achievement and reduced workforce participation rates in adulthood (James et al. 2024). Therefore, assessing these skills with valid and reliable methods at the beginning of schooling is a crucial step toward identifying at-risk children and developing evidence-based preventive interventions.

Assessing early literacy skills goes beyond functioning as a screening tool and serves a strategic role in promoting educational equity. Research indicates that environmental factors such as socioeconomic conditions shape the neurocognitive systems underlying literacy development from an early age (Noble et al. 2021). This highlights the need for assessment instruments that are standardized across all children yet sensitive to cultural and linguistic contexts. A variety of assessment tools are available internationally for evaluating the early literacy skills of preschool children. These tools generally target fundamental components such as phonological awareness, letter knowledge, vocabulary, and reading comprehension. For instance, the STAR Early Literacy Scale comprehensively measures these skills through a computer-adaptive system (Renaissance Institute 2001), whereas the Preschool Early Literacy Indicators (PELIs), developed by Kaminski et al. (2014), assess vocabulary and comprehension skills alongside subdimensions of early literacy, including alphabet and print awareness. Similarly, Barringer (2009) evaluated pre-reading skills such as phonological awareness and grammar using the Early Literacy Knowledge and Skills (ELKS) scale. More specifically, McBride’s (2015) Observation Survey of Early Literacy Achievement scale aims to identify what children already know and what they need to learn at the beginning of the reading and writing acquisition process.

The pace and nature of reading and writing acquisition are significantly influenced by the orthographic depth of the language being learned (Ellis et al. 2004). The transparent orthographic structure of Turkish, characterized by high letter–sound consistency, enables children to acquire decoding skills more rapidly than in languages with opaque orthographies, such as English. This structural difference implies that direct translation or adaptation of tests developed for other languages cannot accurately capture the unique developmental profiles and skill hierarchies of children in Türkiye. Therefore, developing measurement instruments that are specific to Türkiye’s linguistic structure, psychometrically robust, and based on nationally normative data is a critical need. Indeed, although limited, several studies have been conducted in this area. The Early Literacy Skills Assessment Instrument (EOBDA), developed by Karaman and Aytar (2016), assesses phonological awareness, print awareness, story comprehension, visual matching, and prewriting skills among children aged 48–77 months. Similarly, Güven and Topbaş (2014) adapted the Test of Early Language Development (TELD-3) for children in Türkiye.

While a limited number of instruments exist in Türkiye to assess children’s reading readiness within the field of early literacy, these tools remain inadequate due to two fundamental limitations. First, most of them focus on specific pre-reading subskills and fail to comprehensively measure the current proficiency levels of students who have already learned to read and write by the time they enter elementary school—that is, their pre-literacy skills. It is noteworthy that some children in Türkiye begin first grade already knowing how to read and write. However, current random placement practices and standardized phonics instruction often lead to boredom, loss of motivation, and slower progress among these advanced students, thereby reducing the overall effectiveness of instruction. The second and more critical limitation is that many existing local instruments do not meet modern psychometric standards (e.g., testing measurement invariance through multigroup confirmatory factor analysis [MGCFA]). This underscores the need for an instrument that assesses various dimensions of writing skills—particularly those involving both motor and cognitive processes, such as copying and dictation—within a holistic model and rigorously examines their construct validity. In this context, developing a valid and reliable Pre-Literacy Test (PLT) is essential for accurately identifying students’ initial literacy levels upon entering first grade and for providing appropriate placement and individualized instruction, thereby promoting educational equity and supporting individual development. Based on this need, the present study aims to develop the PLT and to comprehensively examine its psychometric properties in order to assess the pre-literacy skills of children transitioning from preschool to elementary school.

The primary objective of this study is to address the critical shortage of literacy assessment tools in Türkiye while challenging the predominantly English-centric paradigm in the international literature. Developed within the context of Turkish transparent orthography, the PLT serves as a pivotal testing ground for the orthographic depth hypothesis and universal theories of literacy acquisition. By providing comparative data from a transparent script, the study aims to offer significant, cross-linguistic evidence that broadens the scope of existing literacy research.

Furthermore, the PLT advances theoretical understanding by operationalizing early writing as distinct cognitive processes—specifically copying and dictation—rather than a unitary construct, offering empirical support for frameworks such as the Not-So-Simple View of Writing and DIEW. Practically, the instrument moves beyond global tools that traditionally focus on precursor skills; instead, it directly measures the holistic proficiencies children demonstrate at school entry. This approach fills a critical gap in the field, enabling educators to look beyond mere readiness to identify specific student needs and implement targeted instruction.

## 2. Materials and Methods

This study employed a quantitative design aimed at developing a measurement instrument to assess the pre-literacy skills of children who have completed preschool and are about to enter first grade. The methodological framework of the study was based on a multistage and comprehensive hierarchy of construct validity and reliability analyses, designed to ensure the psychometric soundness of the scale.

### 2.1. Participants

The study group consisted of children aged 6–7 who were attending preschool preparatory classes and transitioning to first grade in elementary school during the 2024–2025 academic year. Convenience sampling was employed, prioritizing accessibility and suitability for the study’s purpose (Fraenkel Jack et al. 2012). The study was conducted in three subgroups based on the type and stage of analyses. The first study group included 1911 children (49.8% female, 50.2% male); the second comprised 1644 children (47.3% female, 52.7% male); and the third consisted of 2411 children (48.6% female, 51.4% male). Most participants were from families identified as having middle to upper socioeconomic status.

### 2.2. Data Analysis

Data were analyzed using Jamovi 2.6.44. The analyses were conducted in three stages. In the first stage, an Exploratory Factor Analysis (EFA) was performed on data from 1911 participants; in the second stage, a Confirmatory Factor Analysis (CFA) was conducted on data from 1644 participants; and in the third stage, item analyses were carried out on data from 2411 participants. Importantly, each analysis was conducted on a different sample. Using separate samples for EFA, CFA, and item analyses is a methodological requirement to prevent overfitting, ensure the independence of analyses, and increase the generalizability and robustness of the psychometric findings. Therefore, the data for each analytical stage were collected from distinct participant groups.

Before conducting the EFA, the suitability of the data for factor analysis was examined using Bartlett’s Test of Sphericity and the Kaiser–Meyer–Olkin (KMO) Measure Of Sampling Adequacy. After confirming that the sample met the necessary assumptions, an EFA was performed to determine the construct validity of the scale. Principal Axis Factoring (PAF) was employed as the factor extraction method. Assuming intercorrelations among the factors, an oblique Oblimin rotation was applied (Tabachnick and Fidell 2013). To determine the optimal number of factors to be retained, a Parallel Analysis was conducted, as it provides more reliable results (Horn 1965).

CFA were conducted using the Diagonal Weighted Least Squares (DWLS) estimation method, which is appropriate for ordinal data, along with robust standard errors. Because the χ^2^ test tends to be significant in large samples, the evaluation of model fit primarily focused on comparative fit indices: CFI and TLI (≥0.95 indicating excellent fit) and SRMR and RMSEA (≤0.08 indicating good fit) (Hu and Bentler 1999). To demonstrate the structural integrity of the model, convergent validity was tested using item factor loadings (β), Composite Reliability (CR), and Average Variance Extracted (AVE), whereas discriminant validity was assessed using the Fornell–Larcker criterion (Fornell and Larcker 1981). Furthermore, to determine whether the test measured the same construct in the same way across groups, measurement invariance was evaluated across gender using (MGCFA). Following a hierarchical procedure consistent with the ordinal data structure, three sequential models—configural, metric, and scalar—were compared. Establishing scalar invariance ensured the reliability of factor mean comparisons across groups. A ΔCFI value of ≤0.01 was used as the criterion for evaluating model fit between successive models (Cheung and Rensvold 2002).

For item analyses, item difficulty indices were calculated, and item discrimination was evaluated using point-biserial correlation coefficients within the framework of Classical Test Theory. Item difficulty is interpreted as “difficult” as it approaches 0 and “easy” as it approaches 1. Items with high discriminatory power are expected to have point-biserial correlation values above 0.30 (Ebel and Frisbie 1991). Finally, to determine the reliability of the test, Cronbach’s alpha, McDonald’s Omega, Kuder–Richardson 20 (KR-20) coefficients, and split-half reliability values were calculated.

### 2.3. Data Collection Tools

Demographic Information Form. This form was developed to collect information on the participants’ age, gender, and socioeconomic status.

Pre-Literacy Test (PLT). The PLT was developed by a researcher with extensive academic experience—both theoretical and practical—in the field of reading and writing, taking into account the relevant body of literature. The PLT is designed to measure the basic literacy skills that children acquire prior to school entry, whether through formal or informal learning experiences. It consists of two main sections: reading and writing. The reading section comprises five domains that assess children’s phonics, letter knowledge, spelling, word reading, and simple text reading, including a total of six items. The writing section similarly covers five domains with seven items designed to evaluate letter/sound writing, syllable writing, word writing, and simple sentence writing skills (both copying and dictation).

Specific indicators and items (e.g., recognizing or writing specific letters and sounds; reading or writing particular syllables and words; reading or writing simple sentences) were developed to assess student performance in each domain of the PLT. During the test development process, relevant literature on early literacy, literacy development, and educational measurement was reviewed, expert opinions were obtained, and pilot studies were conducted. An Administration Guide was prepared to support the implementation, scoring, and interpretation of the PLT. This guide outlines the expected basic skills, scoring criteria, and sample student responses for each domain.

The PLT consists of three subtests—Reading Skills, Writing Skills (Dictation), and Writing Skills (Copying)—each scored on a 0–4 scale, where 0 = unacceptable, 1 = inadequate, 2 = moderate, 3 = good, and 4 = excellent. The Reading Skills subtest includes six items assessing letter, closed syllable, open syllable, word, sentence, and text reading. For example, in the word-reading item, children are presented with a page containing various words and are instructed to “read the indicated word.” A score of 4 is assigned when the child reads the target word automatically and fluently; a score of 3 when the child reads the word with slight hesitation; a score of 2 when the reading is syllabic or fragmented; a score of 1 when the child attempts to read by sounding out individual letters; and a score of 0 when the child recognizes some letters but cannot read the word either as a whole or in parts. Similarly, in the Writing Skills (Dictation) subtest, children write letters, syllables, words, or sentences dictated by the examiner, while in the Writing Skills (Copying) subtest, they copy visually presented items of the same types. Each response is scored on the same 0–4 scale based on accuracy, completeness, and correctness. For each subtest, the total score is divided by the number of items to obtain a final score between 0 and 4, which is interpreted using the same performance categories (0 = unacceptable, 1 = inadequate, 2 = moderate, 3 = good, 4 = excellent).

### 2.4. Data Collection Process

Ethical approval for the study was obtained from Istanbul University-Cerrahpaşa. Data were collected by classroom teachers working in elementary schools. Prior to data collection, the researcher provided a one-day training program for the teachers, which introduced the instrument and covered the procedures for administration and scoring. In addition, sample application and scoring videos were prepared and shared with the participating teachers.

Data were collected in June and July of 2025, when the children were completing the preschool preparation program and transitioning to first grade. The PLT was administered by classroom teachers in accordance with the procedures outlined in the Administration Guide. Teachers evaluated each child’s reading and writing performance based on their responses to the test items and recorded the scores on the administration form.

## 3. Results

### 3.1. Exploratory Factor Analysis Results

Preliminary analyses conducted to evaluate the suitability of the dataset (*N* = 1911) for factor analysis indicated that the necessary assumptions were met. Bartlett’s Test of Sphericity was statistically significant (χ^2^(78) = 35,606.59, *p* < 0.001), suggesting that the correlation matrix was significantly different from the identity matrix. The KMO Measure of Sampling Adequacy was excellent, with an overall value of 0.945. Additionally, item-level distributional analyses revealed that skewness values ranged from −2.47 to −0.52, and kurtosis values ranged from 5.47 to −0.12, indicating acceptable levels of normality for factor analysis. Taken together, these results confirmed that the data were highly suitable for factor analysis.

PAF was employed as the factor extraction method, and an oblique Oblimin rotation was applied. The number of factors was determined through Parallel Analysis, which indicated a three-factor structure. Factor loadings and covariance values are presented in Table 1, and the explained variance ratios and inter-factor correlations are presented in Table 2.

The three-factor structure obtained from the EFA accounted for 82.38% of the total variance, which is a notably high rate. The factors were named Reading Skills (Factor 1), Writing Skills (Dictation) (Factor 2), and Writing Skills (Copying) (Factor 3), based on the content of the items they comprised. Reading Skills (Factor 1) explained the largest proportion of variance (36.44%), followed by Writing Skills (Dictation) (26.11%) and Writing Skills (Copying) (19.83%). The Pattern Matrix (Table 1) showed that the items loaded clearly and strongly on their respective factors: loadings for the Reading Skills factor (six items) ranged from 0.648 to 0.933; for the Writing Skills (Dictation) factor (four items), from 0.659 to 0.961; and for the Writing Skills (Copying) factor (three items), from 0.765 to 0.927. Furthermore, the communalities (h^2^) for all items ranged from 0.724 to 0.955, confirming that a substantial portion of each item’s variance was successfully explained by the three core skill factors.

The correlations among the factors supported the use of the oblique Oblimin rotation, clearly demonstrating that the factors were highly interrelated. The correlation between Reading Skill and Writing Skill (Dictation) was very high (r = 0.872). Similarly, the correlation between Writing Skill (Dictation) and Writing Skill (Copying) was also high (r = 0.781). Finally, the correlation between Reading Skill and Writing Skill (Copying) was found to be r = 0.631. These strong correlations suggest that the three identified factors may represent subdimensions of a single, unified, yet highly interconnected higher-order construct—reading–writing skills. Following this stage, a CFA was conducted, and the results are presented below.

### 3.2. Confirmatory Factor Analysis Results

The fit indices of the three-factor measurement model obtained from analyses conducted with a dataset of 1644 participants are summarized in Table 3. In addition, item-level distributional analyses indicated that skewness values ranged from −2.41 to −0.41, whereas kurtosis values ranged from 6.24 to −0.11, suggesting that the items demonstrated acceptable levels of normality for confirmatory factor analysis. The model demonstrated excellent fit according to the comparative fit indices. The SRMR value (0.030) was well below the threshold of 0.08, indicating perfect fit. The CFI (0.997) and TLI (0.997) values also exceeded the criterion for excellent fit (≥0.95). However, the scaled RMSEA value was 0.111 (90% CI [0.105, 0.116]), which bordered on poor fit (≤0.08), and the chi-square test, χ^2^(62) = 829.416, *p* < 0.001, was statistically significant. This pattern, often observed in large-sample studies (i.e., high CFI and elevated RMSEA), suggests that although the model demonstrates a strong comparative fit, it does not achieve perfect absolute fit. It is also noted that the DWLS estimation method can inflate RMSEA values (Xia and Yang 2019). To further evaluate the stability of this model fit pattern beyond a purely statistical rationale, we conducted supplementary CFAs using two large independent datasets previously collected for the PLT (Dataset A: EFA sample, *n* = 1911; Dataset B: item analysis sample, *n* = 2411). Across both datasets, the same pattern emerged: CFI/TLI values consistently indicated excellent comparative fit, whereas RMSEA remained moderately elevated (CFI = 0.998, TLI = 0.998, RMSEA = 0.098 for Dataset A; CFI = 0.996, TLI = 0.996, RMSEA = 0.119 for Dataset B).

The model provided strong evidence of convergent validity (Table 4), indicating that the items were highly representative of their respective factors. All standardized factor loadings (β) were statistically significant (*p* < 0.001) and notably high, ranging from 0.905 to 0.992. When examined by factor, the loadings for the Reading Skills items ranged from 0.926 to 0.992, for the Writing Skills (Dictation) items from 0.905 to 0.971, and for the Writing Skill (Copying) items from 0.915 to 0.941. These findings clearly demonstrate that each factor is strongly and accurately represented by its corresponding items.

As summarized in Table 5, the CR (lowest CR = 0.917), Cronbach’s Alpha (lowest α = 0.891), and McDonald’s Omega (lowest ω = 0.912) values calculated for all three factors were well above the acceptable threshold of 0.70. Similarly, the AVE values (lowest = 0.867) also exceeded the criterion of 0.50, indicating strong convergent validity. Examination of the Fornell and Larcker criterion for discriminant validity revealed that the square root of the AVE for each factor was greater than the highest correlation between that factor and the others. Satisfying this condition across all factor pairs demonstrates that, despite strong inter-factor relationships, each construct was sufficiently distinct from the others, thereby confirming discriminant validity.

When the relationships among the factors were examined (Table 5), statistically significant and very high correlations were observed, consistent with the previous EFA findings. The correlation between Reading Skills and Writing Skills (Dictation) was notably strong (β = 0.925, z = 65.441, *p* < 0.001). Similarly, the correlation between Writing Skills (Dictation) and Writing Skills (Copying) was high (β = 0.826, z = 44.203, *p* < 0.001). The correlation between Reading Skills and Writing Skills (Copying) was also substantial (β = 0.674, z = 30.224, *p* < 0.001). These results confirm that the three skill areas represent distinct yet highly interrelated subconstructs.

Finally, measurement invariance of the latent variables was tested across gender groups. As shown in Table 6, interpretation of the Δχ^2^ and ΔCFI values indicated that both metric and scalar invariance levels were achieved. The ΔCFI value was 0.001 for the metric invariance test and 0.001 for the scalar invariance test. These findings confirm that configural, metric, and scalar invariance were established across gender. Attaining scalar invariance demonstrates that comparisons of latent factor means between gender groups are valid.

### 3.3. Item Analysis Results

The item difficulty and item discrimination indices calculated for the 13 questions in the reading–writing test, based on data collected from 2411 participants, are presented in Table 7. Furthermore, item-level distributional analyses showed that skewness values ranged from −2.67 to −0.19, while kurtosis values ranged from 6.27 to −0.25, indicating that the items exhibited acceptable levels of normality for item analysis.

Item analysis results indicate that, although the majority of items in the test were relatively difficult, they exhibited very high quality in terms of discrimination. The discrimination indices of all items exceeded the acceptable threshold of 0.30 (with item 7 being borderline at 0.299). Owing to its high item discrimination power, the test has the potential to consistently and effectively distinguish differences in ability within the measured domain.

The Cronbach Alpha and KR-20 coefficients calculated for the PLT were both 0.768, while the split-half reliability coefficient was 0.854. These internal consistency and reliability values demonstrate performance well above the acceptable threshold, confirming the scale’s reliability.

## 4. Discussion

This study comprehensively examined the psychometric properties of the PLT, which was developed to assess the pre-literacy skills of children transitioning from preschool to elementary school. The key findings provide strong evidence that the PLT is a psychometrically robust, valid, and reliable instrument that not only meets local educational needs but also makes a significant contribution to the international early literacy literature.

One of the most striking findings of the study was the three-factor structure revealed by the EFA and CFA: Reading Skills, Writing Skills (Dictation), and Writing Skills (Copying). This structure supports the general notion that reading and writing are highly correlated yet separable constructs (Kim et al. 2023). Although reading and writing skills are often linked within models of literacy development, recent research suggests that these skills can be differentiated and are associated with at least partially distinct cognitive determinants (Sigmund et al. 2024). The present study’s findings are consistent with this view. Moreover, the current study empirically demonstrated that early writing is not a unidimensional construct. The distinction between dictation and copying as separate dimensions of early writing is increasingly supported by empirical research across diverse orthographies. Recent studies highlight that copying and dictation engage distinct cognitive and motor processes, reinforcing the multidimensional nature of early writing development. Copying tasks primarily depend on transcription-related competencies such as visual perception, fine motor coordination, and visual–motor integration, as demonstrated in both alphabetic and logographic systems (Puranik and Al Otaiba 2012). For example, in Arabic and Chinese, graphomotor and fine-motor skills significantly influence copying performance, while orthographic knowledge is more critical for dictation accuracy (Salameh-Matar et al. 2024; Ye et al. 2021a, 2021b). Dictation, in contrast, requires more complex cognitive processes, including phoneme–grapheme mapping, orthographic knowledge, and auditory short-term memory (Berninger and Winn 2006). Studies in transparent orthographies like Spanish and Italian show that dictation tasks are more sensitive to orthographic complexity and phonological processing demands than copying tasks, and children tend to make more errors in dictation than in copying. This pattern is consistent across languages, suggesting that the cognitive load of dictation is higher due to the need for sound–letter mapping and memory retrieval (Afonso et al. 2020; Arfé et al. 2019).

Theoretical models such as the “Not-So-Simple View of Writing” and the Direct and Indirect Effects Model of Writing (DIEW) emphasize the interplay between lower-order transcription skills (copying, handwriting) and higher-order cognitive–linguistic skills (spelling, phonological awareness) in writing development (Barnett et al. 2019; Kim and Schatschneider 2017). Empirical evidence from cross-linguistic studies further demonstrates that orthographic transparency modulates the relative contribution of these skills, with transparent orthographies placing greater emphasis on phonological processing during dictation (Afonso et al. 2020; Iniesta et al. 2022; Iniesta et al. 2021).

This finding holds critical importance for clinicians and educators, particularly for the early diagnosis of writing difficulties (dysgraphia) and in distinguishing whether the source of the problem lies in motor–coordination or phonological–orthographic processing. Recognizing dictation and copying as distinct dimensions allows for more targeted assessment and intervention. For instance, interventions that separately address graphomotor skills for copying and phonological/orthographic skills for dictation have shown promise in improving early writing outcomes. This nuanced understanding enables educators to better identify specific writing difficulties and tailor instruction accordingly, thereby bridging theoretical insights with classroom practice (Alves et al. 2016; Barnett et al. 2019; McMaster et al. 2017).

The PLT demonstrated strong psychometric properties that align with modern measurement standards. High internal consistency coefficients for both the subscales and the total test indicate that the instrument reliably measures the intended constructs. Convergent and discriminant validity analyses confirmed the structural integrity of the three-factor model based on the Fornell–Larcker criterion. Notably, the high CFI and TLI values observed in the CFA indicate that the proposed model provides an excellent fit to the data. Although the RMSEA value exceeded the conventional threshold—a common occurrence in large samples and with the DWLS estimator—this does not undermine the model’s validity, given the excellent performance of the other fit indices (Shi and Maydeu-Olivares 2020).

One of the most important methodological contributions of the study is the establishment of full scalar measurement invariance across gender groups. This finding demonstrates that the PLT measures the same psychological construct for both boys and girls, and that the items neither advantage nor disadvantage either group (Vandenberg and Lance 2000). Establishing scalar invariance ensures that mean score differences between groups reflect genuine skill differences rather than measurement bias. This enables fair and valid comparisons in research and educational policy aimed at examining gender differences in early literacy skills.

The PLT has important practical implications for the Turkish education system. While traditional early literacy tests often focus on precursor skills (e.g., phonological awareness), the PLT is designed to directly assess the pre-literacy skills that children possess at the beginning of elementary school. Item analysis results show that the test includes both easy and difficult items, with an overall moderate level of difficulty. This feature enables the PLT to effectively identify not only at-risk students who require support but also advanced students who have already learned to read and write. This is a critical step in preventing the “Matthew Effect,” conceptualized by Stanovich (1986), in which strong readers continue to improve while weaker readers fall further behind. Accurately determining students’ initial literacy levels during the first days of school allows teachers to implement differentiated instruction and response-to-intervention models rather than applying a uniform curriculum (Fuchs et al. 2012). This approach prevents advanced students from losing motivation while enabling early and targeted interventions for those who need additional support. In this respect, the PLT stands out as an evidence-based tool that promotes equal opportunity and individualized learning in education.

Beyond classroom practice, the PLT also offers broader practical implications for schools and policymakers. Because the test provides criterion-based and developmentally appropriate information on students’ literacy readiness, schools can use PLT results to plan resource allocation, determine the need for remedial programs, and develop evidence-based literacy initiatives. At the policy level, the PLT can contribute to early screening frameworks, ensuring that literacy difficulties are detected before gaps widen. Furthermore, researchers and curriculum developers can utilize PLT data to monitor cohort-level literacy trends, evaluate the effectiveness of instructional programs, and design interventions tailored to regional or demographic needs. In this respect, the PLT stands out as an evidence-based tool that not only supports individualized learning in classrooms but also informs decision-making processes that promote equity and early prevention in the education system.

## 5. Limitations and Recommendations for Future Research

This study should be interpreted in light of several limitations. First, participants were selected using a convenience sampling method and predominantly came from families with middle- to upper-socioeconomic backgrounds. This sample composition may limit the generalizability of the factor structure, item parameters, and normative characteristics of the PLT. Because socioeconomic context is known to influence early literacy exposure and pre-literacy skills, the current factor structure and item functioning may not fully represent children from lower socioeconomic backgrounds. Therefore, the findings should be interpreted with caution, and overgeneralization should be avoided until the PLT is validated with more socioeconomically diverse samples. Establishing national norms using a larger, more heterogeneous, and representative dataset is an important direction for future research.

Second, although convergent and discriminant validity were examined using statistical indices such as Composite Reliability (CR), Average Variance Extracted (AVE), and the square root of AVE, these analyses did not include external instruments. True convergent and discriminant validity should ideally be assessed using well-established measures in the field. Accordingly, further research is needed to examine how PLT scores relate to other validated pre-literacy and early literacy assessments.

Third, the present study employed a cross-sectional design, and the predictive validity of the PLT—for example, the extent to which PLT scores predict reading achievement at the end of first grade—was not examined. Longitudinal studies are required to determine how effectively the test predicts long-term academic outcomes.

Finally, additional avenues for research should include examining measurement invariance across key demographic and educational groups beyond those included in the current study. Testing invariance across variables such as socioeconomic status, geographical region, and bilingual status would provide stronger evidence for the robustness and fairness of the PLT across diverse populations. Moreover, cross-linguistic comparative studies—particularly within languages that vary in orthographic transparency—would further enrich theoretical models of literacy acquisition and help clarify the extent to which the multidimensional structure of early writing (e.g., copying and dictation) generalizes across orthographies. Such work would both deepen the theoretical contributions of the PLT and enhance its relevance to the broader international literature.

## 6. Conclusions

This study provides strong evidence for the psychometric soundness of the PLT, positioning it as a valid and reliable tool for assessing the pre-literacy skills of children transitioning from preschool to elementary school. Analyses confirmed that the test is supported by a robust three-factor structure encompassing reading, writing (dictation), and writing (copying). This structure elucidates an important distinction in the cognitive processes underlying writing: by empirically differentiating the cognitively demanding dictation component from the visually and motorically driven copying skill, the test offers a refined understanding of the multidimensional nature of early writing development. The instrument’s high internal consistency and full measurement invariance across gender groups demonstrate that the PLT provides a solid methodological foundation for conducting fair and consistent assessments across student populations. Consequently, the PLT extends beyond traditional screening tools by accurately identifying not only children who require support but also those who begin school with advanced literacy skills. In this capacity, the test makes a unique contribution to the international literature by offering a theoretically meaningful and practically applicable assessment framework that supports differentiated instructional practices aimed at mitigating the Matthew Effect.

## Figures and Tables

**Table 1:** Pattern matrix and communalities.

Item	Factor 1	Factor 2	Factor 3	*h* ^2^
Item 1	0.648	0.050	0.150	0.751
Item 2	0.870	0.091	−0.008	0.940
Item 3	0.885	0.073	0.003	0.932
Item 4	0.933	−0.004	0.050	0.942
Item 5	0.898	0.031	0.049	0.955
Item 6	0.689	0.157	0.074	0.864
Item 8	−0.007	0.659	0.250	0.800
Item 9	−0.034	0.961	0.006	0.882
Item 11	0.062	0.911	−0.024	0.903
Item 13	0.163	0.739	0.040	0.860
Item 7	0.073	−0.033	0.791	0.724
Item 10	−0.020	0.016	0.927	0.852
Item 12	0.037	0.110	0.765	0.772
Eigenvalue	9.401	0.755	0.326	
% of Variance	36.436	26.110	19.834	

Note. *N* = 1911. Principal axis factoring with Oblimin rotation was used. *h*^2^ = Communality.

**Table 2:** Factor statistics and correlations.

Factor	Sum of Squared Loadings	% of Variance Explained	Cumulative %
1	4.737	36.436	36.436
2	3.394	26.110	62.545
3	2.578	19.834	82.379
Total	10.709	82.380	
Inter-Factor Correlations			
Factor	1	2	3
Reading skills	0.963α		
Writing skills (dictation)	0.872	0.945α	
Writing skills (copying)	0.631	0.781	0.892α

Note. *N* = 1911. α = Cronbach’s Alpha. α for PLT (Total) = 0.970.

**Table 3:** Model fit indices summary.

Fit Index	Value	Acceptable Threshold	Comment
χ^2^(df)	829.416 (62)	*p* > 0.05	Significant
Scaled RMSEA	0.111, 90% CI [0.105, 0.116]	≤0.08	Poor Fit
Scaled SRMR	0.030	≤0.08	Good Fit
CFI	0.997	≥0.95	Excellent Fit
TLI	0.997	≥0.95	Excellent Fit

Note. *N* = 1644. Estimation method: DWLS, Standard Errors: Robust (Mean adjusted scaled and shifted).

**Table 4:** Measurement model estimates.

Latent Variable	Item	Standardized Loading (*β*)	z	*p*
Reading Skills	i1	0.926	—	—
	i2	0.984	126.80	<0.001
	i3	0.983	133.70	<0.001
	i4	0.988	125.43	<0.001
	i5	0.992	123.85	<0.001
	i6	0.971	122.62	<0.001
Writing Skills (Dictation)	i8	0.905	—	—
	i9	0.948	143.43	<0.001
	i11	0.971	138.85	<0.001
	i13	0.961	132.95	<0.001
Writing Skills (Copying)	i7	0.915	—	—
	i10	0.941	85.35	<0.001
	i12	0.939	85.45	<0.001

**Table 5:** Reliability, convergent, and discriminant validity indices of the factors.

Factor	α	ω	CR	AVE	√AVE	1	2	3
1. Reading Skills	0.962	0.983	0.999	0.949	0.974	—		
2. Writing Skills (Dictation)	0.942	0.955	0.965	0.896	0.947	0.925	—	
3. Writing Skills (Copying)	0.891	0.912	0.917	0.867	0.931	0.674	0.826	—

Note. *N* = 1644. Estimation method: DWLS, Standard Errors: Robust (Mean adjusted scaled and shifted). α for PLT (Total) = 0.966. ω for PLT (Total) = 0.983.

**Table 6:** Measurement invariance results.

		χ^2^	df	CFI	TLI	RMSEA	SRMR	Δχ^2^	Δdf	ΔCFI	*p*
Gender	Configural invariance	864	124	0.997	0.997	0.109	0.031				
	Metric invariance	880	134	0.998	0.998	0.090	0.031	24	10	0.001	0.084
	Scalar invariance	879	170	0.997	0.998	0.092	0.035	01	36	0.001	1.00

**Table 7:** Item difficulty and item discrimination indices.

	Item	Item Difficulty	Point-Biserial
Reading Skills	i1	0.832	0.585
	i2	0.648	0.664
	i3	0.667	0.677
	i4	0.352	0.467
	i5	0.329	0.455
	i6	0.249	0.444
Writing Skills (Dictation)	i8	0.307	0.441
	i9	0.333	0.573
	i11	0.315	0.571
	i13	0.283	0.561
Writing Skills (Copying)	i7	0.310	0.299
	i10	0.361	0.476
	i12	0.335	0.491
Mean Difficulty		0.409	
Mean Discrimination		0.516	

## Data Availability

Restrictions apply to the datasets.

## Abbreviations

The following abbreviations are used in this manuscript:

PLT	Pre-literacy test
EFA	Exploratory factor analysis
CFA	Confirmatory factor analysis
MGCFA	Multigroup confirmatory factor analysis
